# Selectable Ultrasensitive Detection of Hg^2+^ with Rhodamine 6G-Modified Nanoporous Gold Optical Sensor

**DOI:** 10.1038/srep29611

**Published:** 2016-07-12

**Authors:** Zheng Wang, Min Yang, Chao Chen, Ling Zhang, Heping Zeng

**Affiliations:** 1 Shanghai Key Laboratory of Modern Optical System, Engineering Research Center of Optical Instrument and System (Ministry of Education), School of Optical-Electrical and Computer Engineering, University of Shanghai for Science and Technology, Shanghai 200093, China

## Abstract

An extremely sensitive fluorescence sensor has been developed for selectively detection of mercury ions based on metallophilic Hg^2+^-Au^+^ interactions, which results in an effective release of pre-adsorbed rhodamine 6G (R6G) molecules from the nanoporous gold substrate, associated with a significant decrease of fluorescence intensity. The optical sensor has a detection sensitivity down to 0.6 pM for Hg^2+^ and CH_3_Hg^+^ ions, in particular a superior selectivity in a complex aqueous system containing 13 different types of metal ions, meanwhile maintaining a long-term stability after 10 cycles. Such a fluorescence sensor combining multiple advantages therefore present promising potentials in various applications.

Mercury is one of the most toxic pollutant in the environment, which originates not only from the geological events but also from human activities. Volatile mercury is usually oxidized to mercury ions once penetrating into land or water, and then probably enters into the food chain system via microbial biomethylation, which would further cause serious and permanent brain damage and other chronic diseases^1^,^2^,^3^,^4^,^5^. Because of the deleterious effects on human health, development of new mercury detection methods is required to satisfy sensitivity, response, cost, and applicable to different environments. Therefore, sensitive and on-site detection of mercury ions is of paramount importance in environmental protection and healthy monitoring. Current techniques for ion sensing, including atomic absorption spectroscopy (AAS)^6^,^7^ and inductively coupled plasma mass spectrometry (ICP-MS)^8^,^9^ colorimetric and electrochemical methods^10^,^11^, most of them require expensive and sophisticated instrumentation and/or complicated sample preparation procedures. Nondestructive optical detection is an attractive option due to the fast response and available real-time collection in natural environments and living organisms. Accordingly various methods have been developed in the last couple of years, for examples, small organic molecules^12^ (chromophores or fluorophores) and biomolecules (proteins^13^, antibodies^14^, oligonucleotides^15^ and DNAzymes^16^, etc.) functionalized polymeric^17^ and inorganic materials^18^,^19^,^20^,^21^ have been used to construct optical sensor. Although these achievements have proven effective, it is still lack of a method reaching the sensitivity bellow 1 pM which is highly expected in practical applications. Gold nanoparticles based colorimetric and fluorescent sensors^18^,^19^,^20^,^21^,^22^,^23^,^24^,^25^ show excellent selectivity and sensitivity, however, several labels (e.g. DNA) are required to enhance Hg^2+^ ions specificity, which are relatively expensive than common chemical coordinates. It would be of great interest to develop a method combined with several improved characteristics, including sensitivity, selectivity, compatibility (to aqueous environment), cost, speed, simplicity and miniaturize ability.

In this study, we developed a rapid, simple and inexpensive fluorescence method for mercury ions sensing, adopting rhodamine 6G (R6G) and 3-mercaptopropionic acid (MPA) co-modified nanoporous gold (NPG) as the plasmonic substrate. The R6G/MPA NPG fluorescence sensor has a sensitivity of 0.6 pM for both Hg^2+^ and CH_3_Hg^+^ ions detection, which is about 4 orders of magnitude lower than the maximum level of drinking water defined by the United States Environmental Protection Agency (EPA)^26^. Moreover, the R6G/MPA NPG fluorescence sensor exhibits outstanding selectivity to Hg^2+^ ions in both acid and alkaline solutions, and superior stability and anti-interference capacities to other types of different metal ions and even in a natural water system.

## Results

**Figure 1:**
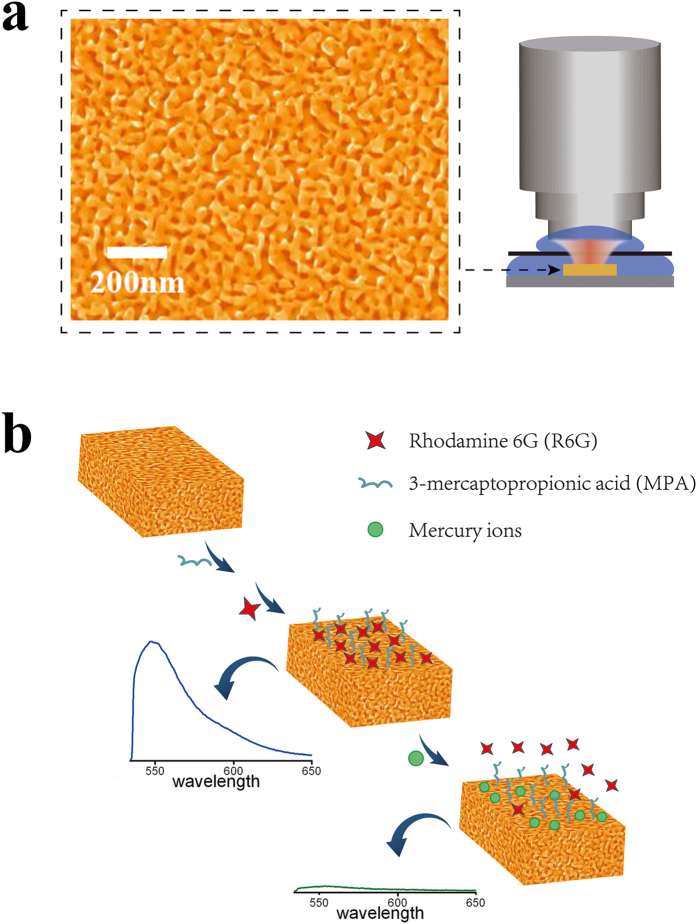
Characterization of the R6G/MPA-NPG fluorescence sensor. (**a**) Typical SEM micrograph of NPG and the experimental set-up for the Hg^2+^ detection. (**b**) Schematic representation of the preparation of the R6G/MPA-NPG fluorescence sensor for detection of mercury ions based on displacement of R6G units on NPG.

NPG with bicontinuous nanopores and ligaments spanning through the entire film, has been demonstrated to be an excellent plasmonic substrate. Due to the large curvature and the electromagnetic coupling between the neighbor ligaments, intense electromagnetic fields can be generated near the nano-scaled gold ligament surface, and fluorescence signal from fluorescent species nearby the NPG surface can be well enhanced^27^. Thus, NPG is a good candidate for sensor applications that require unvarying and reproducible signals from any detected regions of each sample^28^. Figure 1a shows a representative scanning electron microscope (SEM) image of the NPG substrate used in our study. The characteristic length of nanopores is 38 ± 2 nm, measured by a fast Fourier transform method^29^. NPG films are stabilized on polymer sheets for sensing detection, and the schematic of experimental set-up is shown in Fig. 1a.

MPA and R6G are successively assembled onto the gold ligament surface and formed R6G/MPA capped NPG as shown in Fig. 1b. Rhodamine molecule is an extremely fluorescent dye that can noncovalently adsorb onto gold^30^, the emitted fluorescence signal of which can be further enhanced by the NPG substrate ascribed to the localized plasamonic field. However, the interaction between mercury ion and Au is much stronger than that between R6G and Au^31^, and thus the pre-adsorbed R6G would be replaced by mercury ions, accordingly resulting in the dropping of the fluorescence intensity, which is correlated to the concentration of mercury ions^32^. The detection mechanism is generally shown in Fig. 1b.

**Figure 2:**
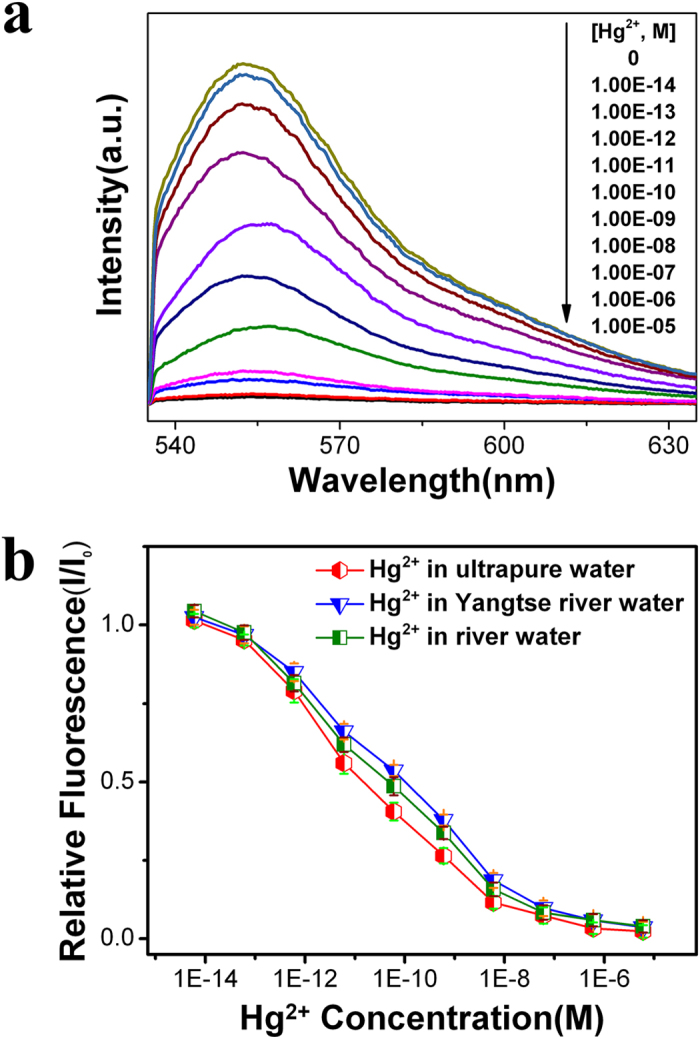
(**a**) Photoemission spectra of R6G/MPA-NPG in the presence of Hg^2+^ with different concentrations. (**b**) Normalized fluorescence intensity variation (I/I_0_) of R6G/MPA-NPG as a function of Hg^2+^ concentration in ultrapure water, Yangtse river water and a tributary of the Huangpu river water. I_0_ is the fluorescence intensity of R6G from the sensor in water only. Excitation wavelength is 532 nm.

Sensitive and quantitative detection of mercury ions is performed by monitoring the intensity changes of R6G fluorescence peaks with mercury ions concentrations. Figure 2a shows the fluorescence spectra that obtained on the NPG film with the ligament size of ~38 nm in diameter, and as shown in figure the fluorescence intensity from R6G reduces with increasing of Hg^2+^ concentration. The normalized fluorescence intensity is given in Fig. 2b. At a low concentration, the number of Hg^2+^ ions is not sufficient to replace all the pre-absorbed R6G molecules, and some R6G molecules are still noncovalent binding to the NPG surface. Thus, the fluorescence signals only partially decrease. With the increasing adding of Hg^2+^ to 10^−8^ M, almost all the R6G molecule have been “washed away” by the mercury ions, and really weak fluorescence signal can be detected. Generally, the fluorescence intensity of the R6G/MPA-NPG sensor toward Hg^2+^ concentration declined almost linearly to the Hg^2+^ concentration, and both the detection limit and the dynamic detection range vary with the sizes of the nanopore and ligament of NPG (see Fig. S1 in Supplementary Information). For the NPG with ligament size of ~22 nm, the detective sensitivity is around 10^−8^ M with a 3 orders of magnitude dynamic range. The detective sensitivity increases with the growing of the ligament size and reaches the ultimate value of 10^−13^ M for the ligament size of about 38 nm, which results in the optimal detection limit of sub-ppt (0.6 pM) for Hg^2+^. The detection sensitivity seldom changed when we replaced the laboratory ultrapure water by river water (the Yangtse river water and a tributary of the Huangpu river water), and the decrease of the fluorescence intensity remains at ~20% with 0.6 pM Hg^2+^ adding in the two kinds of river water. Moreover, the R6G/MPA-NPG sensor does not obvious response to the pH variation (pH value from 4 to 10), and the detective limit is about 10^−12^ M in alkaline solution and 10^−11^ M in acid solution (see Fig. S2 in Supplementary Information).

**Figure 3:**
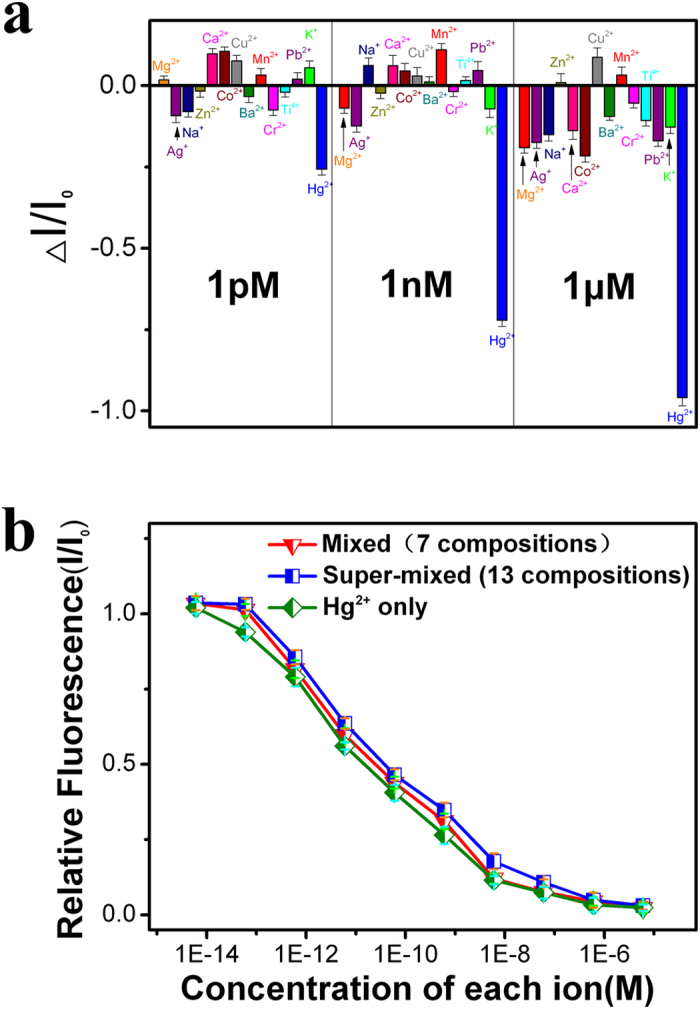
Selectivity test of the R6G/MPA-NPG sensor. (**a**) Normalized fluorescence change of R6G with individual metal ions at various concentrations. (**b**) The normalized fluorescence intensities with various mixed ions at different concentrations. The rhomb dots indicate the solution only contains Hg^2+^. The inverted triangle dots (mixed) indicate the solution contains Hg^2+^, Ag^+^, Mn^2+^, Mg^2+^, Ca^2+^, K^+^, and Ba^2+^ with the same concentrations, and the square dots (super-mixed) indicate the solution contains Hg^2+^, Ag^+^, Mn^2+^, Mg^2+^, Ca^2+^, K^+^, Ba^2+^, Cu^2+^, Cr^2+^, Na^+^, Co^2+^, Zn^2+^, and Pb^2+^ with the same concentration. Excitation wavelength is 532 nm.

The high specificity of Hg^2+^-Au^+^ interactions provided the excellent selectivity of this method towards detecting Hg^2+^ in river water which may contains several environmentally relevant metal ions^31^. To confirm the selectivity of the sensor, other 13 different metal ions (including Mg^2+^, Ag^+^, Na^+^, Zn^2+^, Ca^2+^, Co^2+^, Cu^2+^, Ba^2+^, Mn^2+^, Cr^2+^, Ti^4+^, Pb^2+^ and K^+^ ions) with the same concentrations (1 μM, 1 nM and 1 pM) of Hg^2+^ were separately added into the water, and the response of the fluorescence sensor is depicted in Fig. 3a. As shown in the figure, the sensor shows appreciable intensity change in the response to Hg^2+^. The more Hg^2+^ ions are introduced into the solution, the lower the fluorescence intensity is. For Mg^2+^, Na^+^, Zn^2+^, Ca^2+^, Co^2+^, Cu^2+^, Ba^2+^, Mn^2+^, Cr^2+^, Ti^4+^, Pb^2+^, and K^+^, within the dynamic range (1 nM and 1 pM) the fluorescence intensity does not change perceptibly (less than ±10%). The fluorescence intensities slightly fluctuate with the adding of the other 13 kinds of metal ion (including Mg^2+^, Ag^+^, Na^+^, Zn^2+^, Ca^2+^, Co^2+^, Cu^2+^, Ba^2+^, Mn^2+^, Cr^2+^, Ti^4+^, Pb^2+^, and K^+^ ions). However, even at 1 μM higher concentration, the largest change of the intensity is around 20%, where Hg^2+^ ions led to almost 100% dropping of R6G/MPA-NPG fluorescence. The intensity decreases slightly for Ag^2+^, but the variation less than 20% even with a concentration of 1 μM.

A substitutive characteristic of the selectivity of a sensor is not only detection of isolated targets but also to mixtures that are similar to the natural environments in on-site analysis. We first mixed Hg^2+^ with six other metal ions (Mn^2+^, Ag^+^, Mg^2+^, Ca^2+^, K^+^, and Ba^+^) that are weakly response to the sensor during individual measurements. As shown in Fig. 3b (inverted triangle dots), the interference from the six types of metal cations only gives rise to a slight decrease in the sensitivity, and about 19% reduction still remains in the presence of 1 pM Hg^2+^. Accordingly, we further added six more cations (Cu^2+^, Cr^2+^, Na^+^, Co^2+^, Zn^2+^, and Pb^2+^) into the solution, and each of them has the same concentrations as Hg^2+^. The reduction of the R6G fluorescence intensity, corresponding to 1 pM Hg^2+^, can still keep at approximately ~15% even in such a complex solution (Fig. 3b square dots). As shown in Fig. 3b, three dot lines (inverted triangle dots, square dots and rhomb dots) decrease with the concentration of Hg^2+^ in similar gradient, suggesting that other metal ions produced little influence on mercury ion detection with R6G/MPA-NPG sensor.

**Figure 4:**
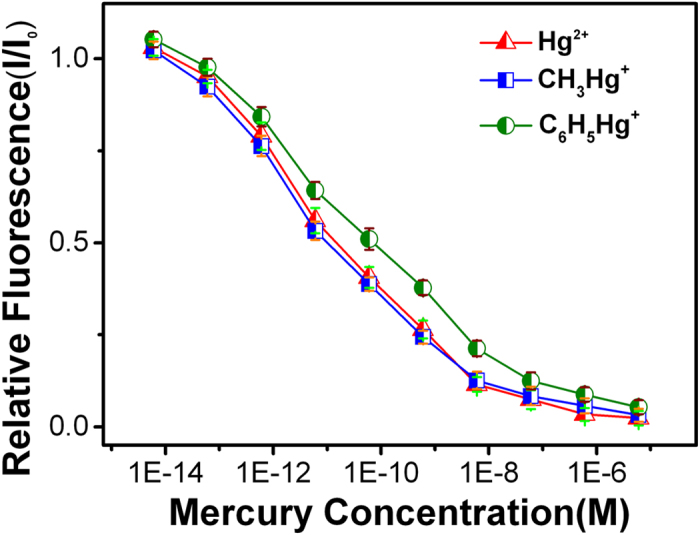
Normalized fluorescence intensity variation (I/I_0_) of R6G/MPA-NPG as a function of mercury ion (Hg^2+^, CH_3_Hg^+^ and C_6_H_5_Hg^+^) concentration.

The sensor not only available for detection of inorganic mercury ions, but also can be used to detect organomercury. Two kinds of organomercury, methylmercury (CH_3_Hg^+^) and phenylmercury (C_6_H_5_Hg^+^), are chosen for the detection. As shown in Fig. 4, the fluorescence variations of three specific mercury species are similar. In general, the curve of fluorescence intensity toward C_6_H_5_Hg^+^ concentration has better linearity, and the sensitivity for detecting methylmercury even better than Hg^2+^, probably due to the better binding ability of methylmercury with carboxyl of MPA on the NPG surface.

**Figure 5:**
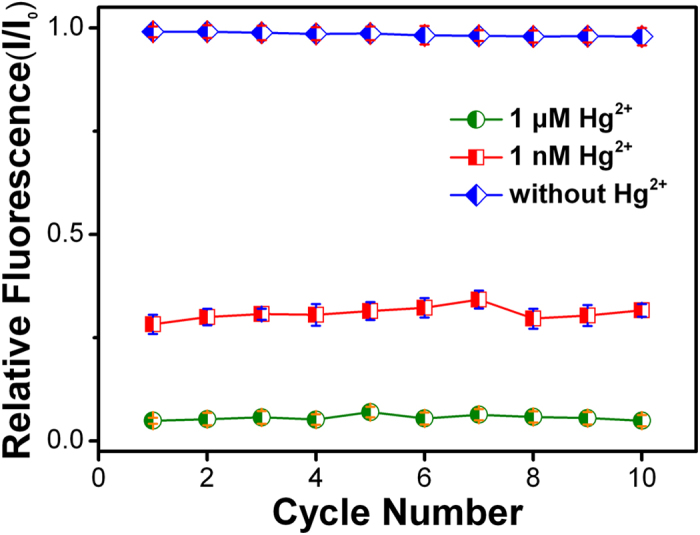
Recyclability test of the R6G/MPA-NPG sensor. Normalized fluorescence peak intensities with 1 nM (square dots) or 1 μM (round dots) Hg^2+^ in the aqueous solution for 10 cycles. I_0_ indicates the fluorescence intensity of the R6G/MPA-NPG sensor before the recyclability test.

Reversibility and regeneration are the most important factor for the sensor, especially in remote and on-site applications. The R6G/MPA-NPG fluorescence sensor can be reused for several times and easily regenerated. After carefully wash with distilled water, the sensor can be easily reprocessed by immerging in 1 mM 3-mercaptopropionic acid (MPA) solution for 1 h, and then in R6G solution for 2 h. Figure 5 indicates 10 normative cycles of the regeneration of the R6G/MPA-NPG fluorescence sensor for detecting 1 nM and 1 μM Hg^2+^ in aqueous solutions. It can be seen that the fluorescence intensity decreases to 28–32% of the original one with 1 nM Hg^2+^, while 1 μM Hg^2+^ produces more than 93% decrease of the original intensity. Although the relative intensity of the fluorescence signal from R6G cannot be fully recovered, the variation ratio is less than 3% within 10 cycles.

## Discussion and Conclusions

Compared with the previous works about mercury detection based on fluorescent molecule and/or novel metal nanoparticles, our method is much simpler and presents higher sensitivity. In order to prevent or induce the aggregation of the nanoparticles, additional proteins (e.g. SBA) or chelating ligands or DNA sequences are popularly used to cap the particles, which results in more complicated manipulation procedures and higher cost. Moreover, the detective sensitivity is kept within ppb-level, and selectively sensitive to either organic or inorganic mercury ions. By using R6G/MPA-NPG fluorescence sensor, only common dye molecules and one traditional chemicals are used to modified the NPG surface, and the limit of detection for both inorganic mercury ions(Hg^2+^) and organic mercury ions(CH_3_Hg^+^ and C_6_H_5_Hg^+^) are ppt-level.

In conclusion, we developed a R6G/MPA-NPG fluorescence sensor to detect mercury ions with very high selectivity and sensitivity. The sensing mechanism is based on the high-affinity metallophilic Hg^2+^-Au^+^ interactions, which results in an effective decrease of the fluorescence intensity of pre-absorbed R6G. The R6G/MPA-NPG fluorescence sensor showed a high selectivity for mercury ions over other metal ions, even at the concentration down to 1 pM (0.2 ng/L). Compared to the traditional Atomic Absorption Spectroscopy (detection limit is 0.01 μg/L) and cold atomic absorption spectrophotometry (detection limit is 0.1 μg/L) methods that used for Hg^2+^ evaluation, the R6G/MPA-NPG fluorescence method is more than hundred times sensitive. Also, this process is worth to be noticed as it involves green chemistry, and could be developed as a simple tool to detect dilute Hg^2+^ pollutions in water and foods for environmental and health monitoring.

## Methods

### Materials

MPA, R6G were purchased from Aladdin (Shanghai). Nitric acid and all of the metal salts used in this study were obtained from Sinopharm Chemical Reagent. Ultrapure deionized water was employed to prepare all the solution freshly and was used immediately. Two kinds of water samples were collected from the Yangtse River and a tributary of the Huangpu River.

### Nanoporous Gold Films Preparation

The 100 nm thick NPG (Nanoporous Gold) films with pore sizes of ~38 nm were prepared by selective dissolution of silver from Au_35_Ag_65_ (atom %) alloy leaves using 71% nitric acid at room temperature^33^,^34^,^35^, for 6 h. After being carefully washed with ultrapure water, the as-prepared NPG films were physically attached to the Polymer Substrate (PS) and heated at 80 °C for 4 h to strengthen the bonding between NPG and PS. At this temperature, both PS and NPG films are very stable, and detectable volume contraction of PS and nanopore coarsening cannot be found^27^. Microstructure of the NPG films was characterized by using a scanning electron microscope (SEM: FEI, Quanta 3D FEG).

### R6G/MPA-NPG hybrid optical sensor

The NPG film was modified with an aliquot of MPA (1 mM) solution for 1 h to stabilize MPA molecules on NPG ligaments. After washed with distilled water, the MPA-NPG composites were immersed in a R6G solution (0.1 μM) for 2 h to further modify fluorophore on the hybrid sensor^36^. The optical sensor was dried in the air kept in drying cabinet. For the metal ions detection, the optical sensor was immersed by metal ions for 15 min at room temperature, and then transferred to pure water container for *in situ* fluorescence measurements using the water immersed objective lens. Each fluorescence spectrum was averaged by the fluorescence spectra collected from ten sites of the substrate.

### Fluorescence Spectroscopy

A home-made fluorescence detection system was used for fluorescence measurement. A micro-fiber spectrometer (Ideaoptics, NOVA) was combined with microscope to collect the fluorescence signal, and 532 nm laser was adopted for the excitation. The laser power was 1 mW at the sample surface, and a water immersed objective lens (Nikon, 60×/1.20 W, WD 0.31–0.28) was used for *in situ* measurements in aqueous solutions.

## Additional Information

**How to cite this article**: Wang, Z. *et al*. Selectable Ultrasensitive Detection of Hg^2+^ with Rhodamine 6G-Modified Nanoporous Gold Optical Sensor. *Sci. Rep.* **6**, 29611; doi: 10.1038/srep29611 (2016).

## Supplementary Material

Supplementary Information
