# Supplementary material for: Selectable Ultrasensitive Detection of Hg2+ with Rhodamine 6G-Modified Nanoporous Gold Optical Sensor
Source: Sci Rep. 2016 Jul 12;6:29611. doi: 10.1038/srep29611 (PMC4940742; doi:10.1038/srep29611)
Supplement: Supplementary Information [file srep29611-s1.pdf]

## SUPPLEMENTARY INFORMATION

# Selectable Ultrasensitive Detection of $Hg^{2+}$ with Rhodamine 6G-Modified Nanoporous Gold Optical Sensor

Zheng Wang, Min Yang, Chao Chen, Ling Zhang* and Heping Zeng*

Shanghai Key Laboratory of Modern Optical System, Engineering Research Center of Optical Instrument and System (Ministry of Education), School of Optical-Electrical and Computer Engineering, University of Shanghai for Science and Technology, Shanghai 200093, China.

*e-mail: lzhang@usst.edu.cn; hpzeng@phy.ecnu.edu.cn

## SUPPLEMENTARY FIGURES

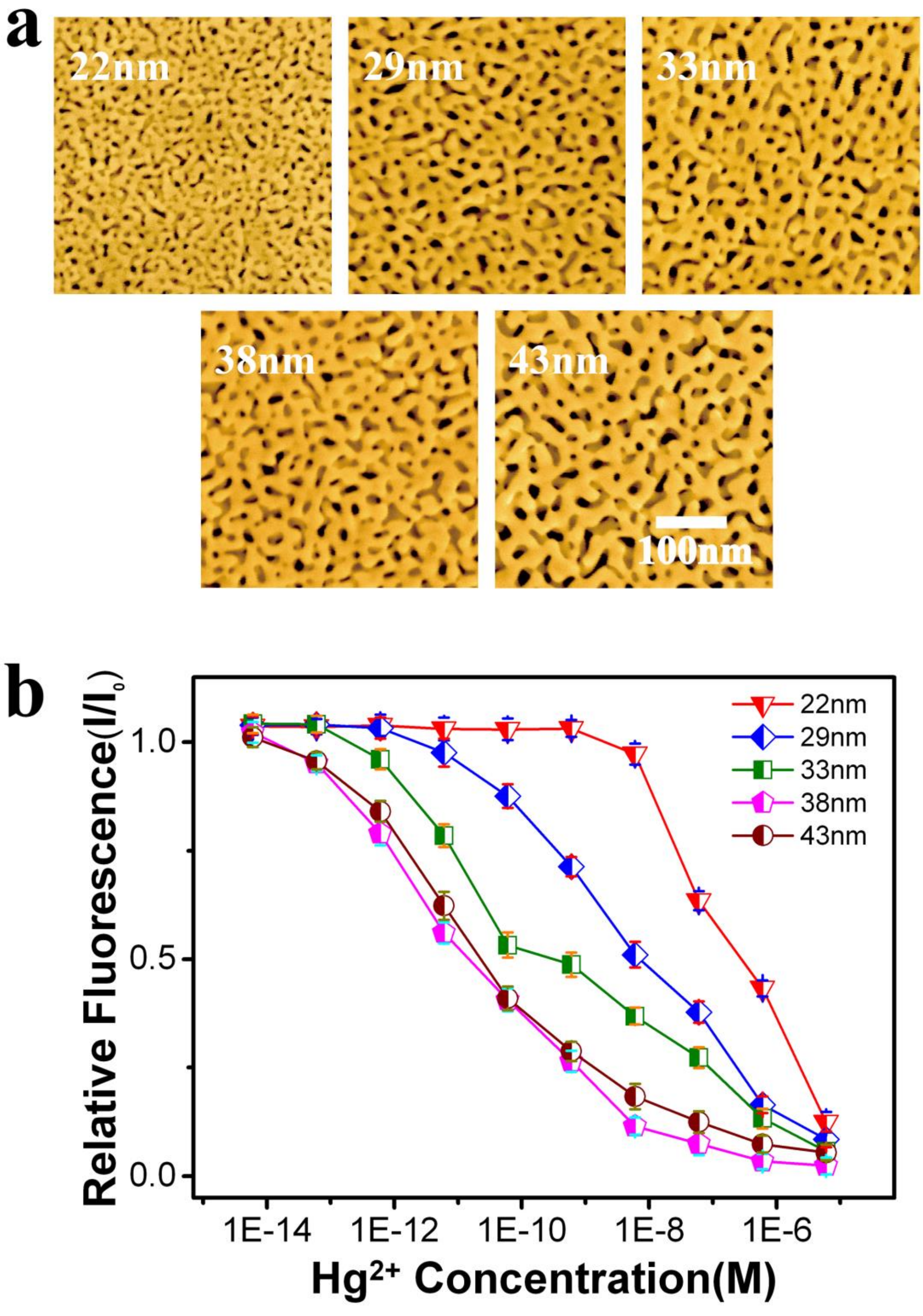


**Figure S1** The nanopore size effect test of the R6G/MPA-NPG sensor. (a) Normalized fluorescence intensity variation ($I/I_0$) of R6G/MPA-NPG with different apertures. (b) The SEM micrograph of NPG used in this test. The nanopore size of NPG from left to right is 38nm, 43nm, 33nm, 29nm, 22nm, respectively.

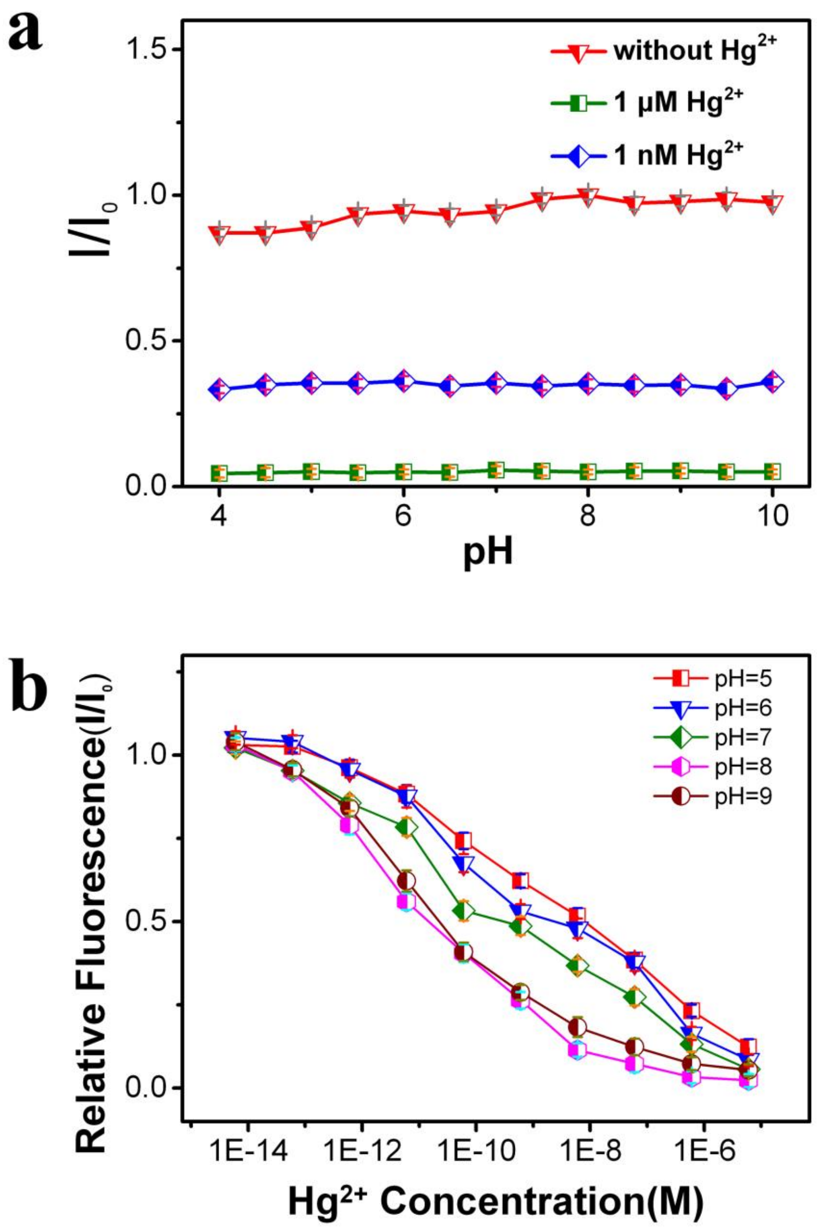


**Figure S2** pH dependent fluorescence response of the R6G/MPA-NPG sensor. (a) The triangle dots indicate the normalized fluorescence intensity variation ($I/I_0$) of R6G/MPA-NPG. The rhombus dots and the hexagon dots indicate the normalized fluorescence intensity variation ($I/I_0$) of R6G/MPA-NPG with the addition of $Hg^{2+}$ (1nM and 1μM, respectively). Excitation wavelength is 532nm. (b) Normalized fluorescence intensity variation ($I/I_0$) of R6G/MPA-NPG in the presence of different pH value (5-9) as a function of $Hg^{2+}$ concentration. $I_0$ indicates the fluorescence intensity of the R6G/MPA-NPG sensor before the recyclability test (pH =7).
